# Sinus arrest in a p.Arg160X-DSP-positive patient without evidence of desmoplakin-mediated cardiomyopathy: a case report

**DOI:** 10.3389/fcvm.2023.1328898

**Published:** 2023-12-19

**Authors:** Nicholas Y. Tan, John R. Giudicessi, Jason R. Harvey, Samuel J. Asirvatham, Konstantinos C. Siontis

**Affiliations:** Department of Cardiovascular Medicine, Mayo Clinic Rochester, Rochester, MN, United States

**Keywords:** sinus node dysfunction, sinus arrest, syncope, desmoplakin, arrhythmogenic cardiomyopathy

## Abstract

**Background:**

Pathogenic/Likely pathogenic variants in *DSP*-encoded desmoplakin are strongly associated with arrhythmogenic cardiomyopathy (ACM). However, their contribution towards sinus node dysfunction has not been well-delineated.

**Case summary:**

A 74-year-old man with a pathogenic variant of *DSP*-encoded desmoplakin (c.478C >T; p.Arg160X) but no evidence of ACM presented with one episode of syncope in the setting of a gastrointestinal illness. Workup including echocardiography, cardiac magnetic resonance imaging, and Holter monitor did not show evidence of ACM or significant arrhythmias. One month later, he experienced several closely-spaced episodes of syncope associated with long sinus pauses and sinus arrest documented on telemetry. He underwent urgent dual chamber pacemaker implantation, during which a ventricular programmed stimulation study was performed and was negative for sustained ventricular arrhythmias. His syncopal episodes resolved and he had no recurrent events on three-month follow-up.

**Discussion:**

As highlighted here, *DSP*-encoded desmoplakin pathogenic/Likely pathogenic variants may contribute to isolated sinus node dysfunction. This clinical link should be further explored in larger studies involving patients with *DSP* variants.

## Introduction

Desmoplakin (DSP) plays a key role in maintaining cell-cell contact through its tethering of intermediate filaments to cardiac desmosomes (1, 2). Pathogenic/Likely pathogenic (P/LP) variants in *DSP*-encoded desmoplakin most commonly result in a distinct form of arrhythmogenic cardiomyopathy (ACM) characterized by left greater than right ventricular (RV) dysfunction, prominent subepicardial delayed enhancement on cardiac magnetic resonance imaging (cMRI), and episodic myocardial injury (3). However, the role of desmoplakin in regulating sinus node activity has not been fully elucidated. In this report, we highlight a case of isolated symptomatic sinus node dysfunction (SND) in a p.Arg160X-DSP-positive patient.

## Case presentation

**Figure 1:**
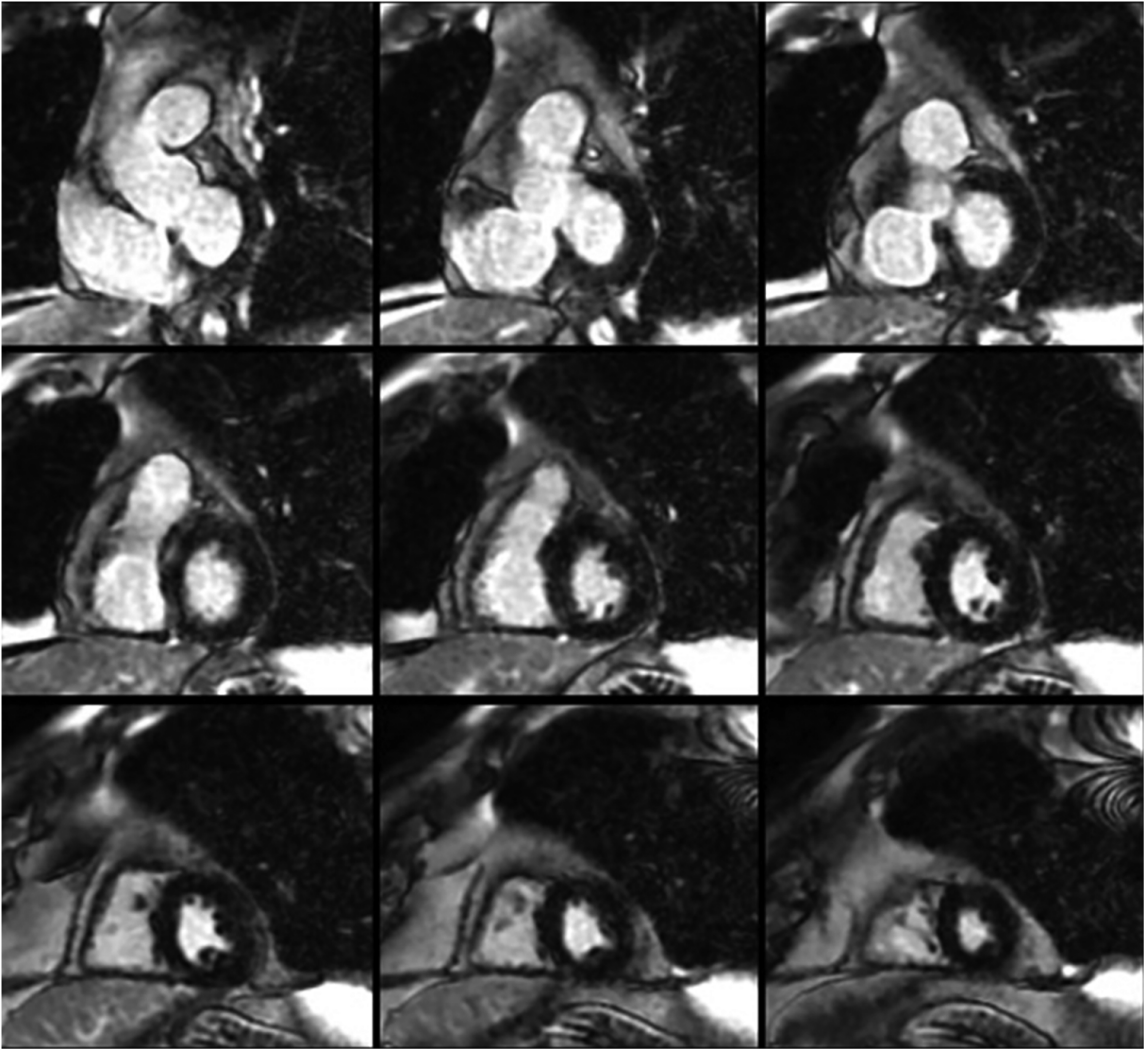
Short-axis cardiac magnetic resonance images demonstrating normal biventricular size and no evidence of late gadolinium enhancement.

**Figure 2:**
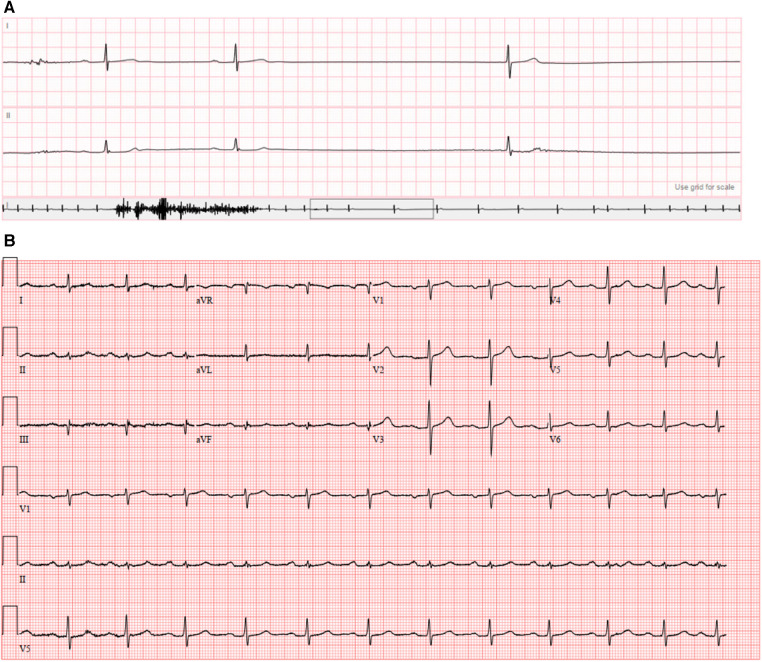
(**A**) Event monitor demonstrating sinus pause of 3.7 s; (**B**) presenting 12-lead electrocardiogram.

A 74-year-old man presented with multiple episodes of syncope. One episode occurred while he was operating a skid steer and three others occurred while he was seated in his chair. Each episode was associated with brief prodromal symptoms of lightheadedness and chest discomfort. He regained consciousness within a few seconds after each event; no seizure-like activity or loss of bowel/bladder control were noted. His background history was significant for a pathogenic variant in *DSP*-encoded desmoplakin (c.478C >T; p.Arg160X). The mutation had been identified one year prior via variant-specific cascade screening because his nephew was diagnosed with DSP-mediated ACM after a resuscitated cardiac arrest. At the time, the patient underwent comprehensive workup including electrocardiogram, echocardiogram, and cMRI which were normal (Figure 1); no evidence of ACM or arrhythmias was found. Left atrial volume index was normal (19 ml/m^2^) and right atrial size was visually normal on echocardiography. There was no family history of sinus node dysfunction or bradycardias. Approximately one month prior to the current presentation, he had completed an event monitor after experiencing a syncopal episode in the context of a gastrointestinal illness. The event monitor showed several nocturnal pauses up to 3.7 s in duration (Figure 2A) without corresponding symptoms. He was not taking any atrioventricular nodal blocking or antiarrhythmic medications at the time of the event monitor or during his current presentation.

**Figure 3:**
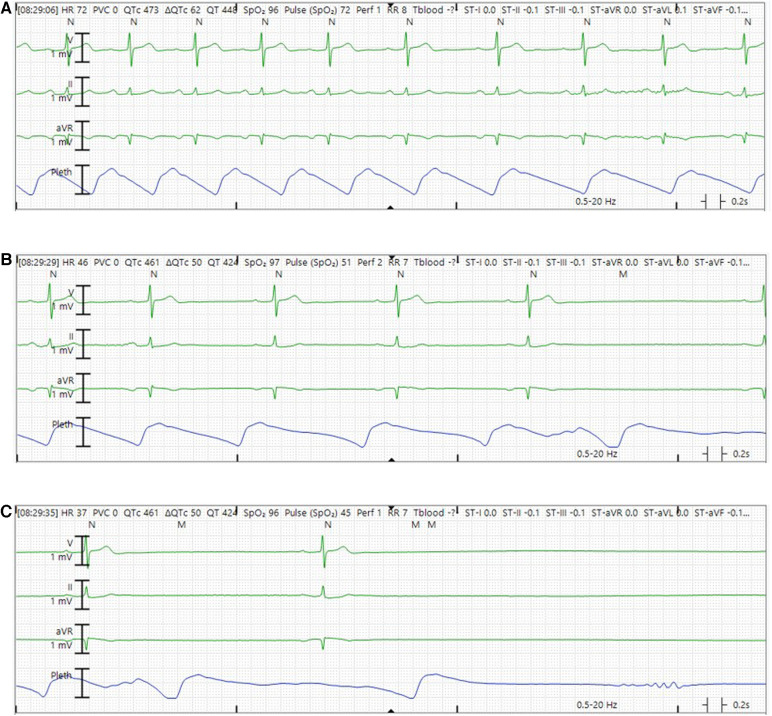
Representative telemetry tracings demonstrating sinus arrest episode while in the hospital. (**A**) Sinus rhythm with rate slowing in the middle of the tracing. (**B**) Sinus bradycardia with further slowing of sinus rate. (**C**) Marked sinus bradycardia before frank sinus arrest.

The patient was vitally stable on presentation (heart rate 74 beats per minute, blood pressure 127/77 mmHg, temperature 36.8°C, respiratory rate 14/min, and oxygen saturation 97%). Blood work including complete blood count, basic metabolic panel, and troponin were normal. His electrocardiogram (Figure 2B) showed sinus rhythm with first degree atrioventricular block (PR interval 230 ms). He was admitted to the cardiac intensive care unit and monitored on telemetry. Shortly afterwards, he had several episodes of marked sinus rate slowing followed by overt sinus arrest up to 8 seconds in duration (Figure 3). His previously described symptoms were reproduced consistently during these events.

**Figure 4:**
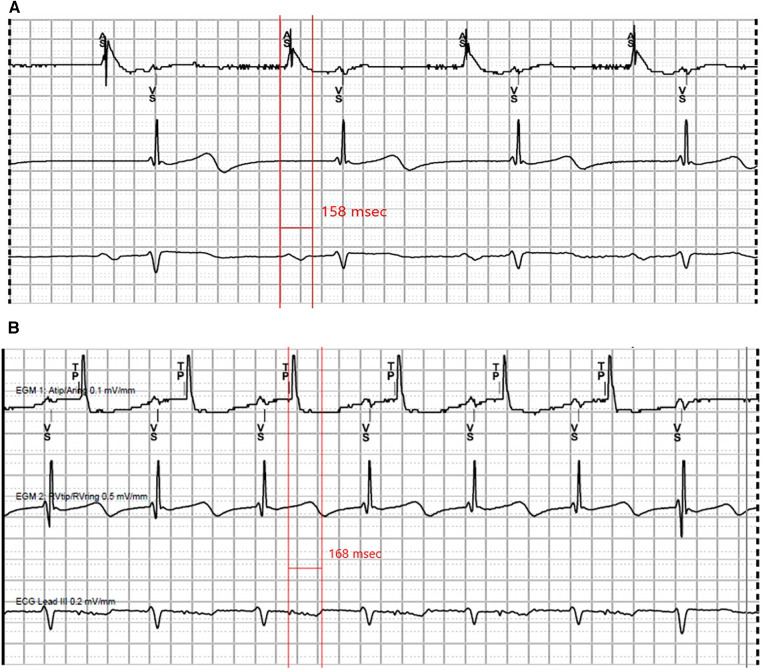
Representative tracings recorded at pacemaker implant. (**A**) Intrinsic sinus rhythm, P wave measuring 158 ms. (**B**) Atrial pacing at 500 ms, P wave measuring 168 ms.

Due to the frequent and concerning nature of his episodes, he underwent urgent dual chamber pacemaker implantation. During the procedure, because of his DSP-positive status, ventricular programmed stimulation was performed via the RV pacing lead which did not induce sustained ventricular arrhythmias; hence, an implantable cardioverter-defibrillator was deferred. Atrial pacing with the lead positioned in the right atrial appendage demonstrated fragmented, low-amplitude and prolonged paced P wave suggestive of significant intra-atrial conduction delay and atriopathy (Figure 4). AV nodal Wenckebach was seen with atrial pacing at 150 beats per minute. His syncopal episodes resolved after pacemaker implantation with no recurrent events at three-month follow-up. Atrial and ventricular pacing percentages were 39.1% and 4.98% respectively, with the device programmed AAI<->DDD with lower pacing rate set at 60 beats per minute.

## Discussion

In this report, we describe a patient with a p.Arg160X-DSP pathogenic variant without imaging evidence of ventricular cardiomyopathy who experienced acute onset recurrent syncope due to episodic sinus arrest suggestive of severe SND. To our knowledge, isolated symptomatic SND associated with pathogenic/Likely pathogenic *DSP* variants has not been reported previously.

*DSP* genotype-phenotype correlations have been best investigated in the context of ventricular arrhythmias and dysfunction. Initially, pathogenic/Likely pathogenic variants in *DSP*-encoded desmoplakin were observed in patients with arrhythmogenic right ventricular cardiomyopathy (ARVC) (4). However, recent studies have demonstrated that pathogenic/Likely pathogenic *DSP* variants more commonly result in a unique form of arrhythmogenic left ventricular cardiomyopathy (ALVC) or biventricular ACM characterized by recurrent acute myocardial injury, LV-predominant involvement, and increased risk of ventricular arrhythmias (3, 5, 6). Although less well-studied, *DSP* may play an important role in the sinus node. Using a novel cardiac conduction-specific *DSP* knockout (KO) mouse model, Mezzano et al. demonstrated that elimination of *DSP* expression in the sinus node led to loss of desmosomal proteins and structures (7). Interestingly, increased sinus pauses and inter-beat variability were observed compared to controls. Furthermore, the same investigators also reported a case of a 19-year-old male heterozygous for a pathogenic *DSP* splice site variant (c273+5G>A, IVS2+5G>A) who exhibited frequent asymptomatic sinus pauses but no evidence of ventricular arrhythmias or systolic dysfunction.

Similarly, the pathogenic *DSP* variant observed in our report is a nonsense mutation that is predicted to be protein-truncating (1, 8). Although p.Arg160X-DSP has been associated with both ACM and dilated cardiomyopathy in the literature (3, 4, 8, 9), SND has not been consistently observed in individuals positive for p.Arg160X-DSP or other *DSP* truncating variants in general. Even so, Mezzano et al.'s findings provide biological plausibility that *DSP* P/LP variants can lead to bradyarrhythmias especially when the sinus node is predominantly implicated. However, this mechanism should be replicated and investigated in greater detail before more definitive conclusions can be made.

We highlight some limitations of the presented case. First, we cannot rule out the possibility of age-related sinus node degeneration given that our patient was relatively older. However, the low-amplitude, fragmented and prolonged P waves are suggestive of a concomitant atrial myopathy. The underlying pathophysiology of atriopathy in a patient with deficient DSP may also include intercellular conduction slowing due to gap junction dysfunction within the sinus node as well as the sinoatrial interface (7). These additional pathologic features increase suspicion for the *DSP* pathogenic variant, rather than age, being the primary driver of his conduction abnormalities, though further study of the association and underlying mechanisms of SND in the setting of desmoplakinopathy is required. Second, electroanatomic mapping would be required to more definitively assess intra-atrial activation times and anatomic sites of conduction delay. Third, although there has been no evidence of ventricular involvement thus far, ventricular electropathy preceding cMRI-detected fibrosis is possible as has been described in ARVC (10). Finally, we observed a small degree of ventricular pacing on follow-up device interrogation. This may be due to intermittent block at the level of the atrium and/or atrioventricular node. Although a full electrophysiology study was not performed, he had not manifested with high-grade atrioventricular block on his event monitor, making infrahisian block a less likely mechanism for this finding.

In conclusion, *DSP* variants may be associated with isolated SND even in the absence of ventricular arrhythmias or cardiomyopathy. Further investigation of this possible link and underlying pathogenetic mechanisms should be performed in large cohorts of patients with *DSP* variants.

## Patient perspective

The patient expressed his willingness and interest to have his case shared with the medical community so that our understanding regarding his genetic mutation and associated issues can be increased. As such, he consented to have the case written and published by the manuscript authors as per COPE guidelines.

## Data Availability

The original contributions presented in the study are included in the article/Supplementary Material, further inquiries can be directed to the corresponding author.
